# Spatial and Temperature Resolutions of Magnetic Nanoparticle Temperature Imaging with a Scanning Magnetic Particle Spectrometer

**DOI:** 10.3390/nano8110866

**Published:** 2018-10-23

**Authors:** Jing Zhong, Meinhard Schilling, Frank Ludwig

**Affiliations:** Institute for Electrical Measurement Science and Fundamental Electrical Engineering, TU Braunschweig, 38106 Braunschweig, Germany; m.schilling@tu-bs.de (M.S.); f.ludwig@tu-bs.de (F.L.)

**Keywords:** magnetic nanoparticles, temperature imaging, scanning magnetic particle spectrometer, spatial resolution, and temperature resolution

## Abstract

This paper quantitatively investigates the spatial and temperature resolutions of magnetic nanoparticle (MNP) temperature imaging with a multiline phantom filled with MNPs. The multiline phantom in total consists of seven lines with different distances between two adjacent lines. A scanning magnetic particle spectrometer is used to measure the spatial distributions of the MNP harmonics for MNP concentration and temperature imaging, whereas an iterative deconvolution method is used to improve the spatial resolution. A modulation transfer function calculated from the MNP concentration image is used to quantitatively present the spatial resolution, whereas the standard deviation of the measured temperatures is used to quantitatively present the temperature resolution. The spatial resolution is about 4 mm while the temperature resolution is about 1.0 K without deconvolution. With increasing the number of the iterative loops in the deconvolution, the spatial resolution is improved to 2 mm while the temperature resolution is worsened to about 9.6 K due to deconvolution-based oscillation.

## 1. Introduction

Temperature is one of the most important parameters affecting the biological and physiological status of a human body. Temperature determination plays a significant role in disease diagnostics and treatment. For instance, infrared thermography is used to provide information on breast cancer [1,2]. Temperature is one of the key factors affecting the treatment efficiency of cancer with magnetic hyperthermia [3,4]. Noninvasive and in-vivo temperature determination contributes to construct a temperature-controlled magnetic hyperthermia system for cancer therapy. In addition, a novel tool of in-vivo temperature determination is crucial to thermally controlled drug delivery for in-situ and quantitative drug release [5,6,7]. Therefore, a novel method of noninvasive and in-vivo temperature imaging is highly relevant to biomedical and biological applications.

However, accurate assessment of in-vivo temperature is still a challenging and hot research topic. Lots of efforts have been made in noninvasive temperature imaging beneath the surface of an object. Recently, magnetic nanoparticles (MNPs) have been used as temperature sensors for noninvasive temperature measurement based on the temperature sensitivity of MNP magnetization induced by magnetic fields. In a direct current (DC) magnetic field, the static magnetization versus applied DC magnetic field *M*(*H*) curve is measured to calculate temperature [8,9,10]. In an alternating current (AC) magnetic field, the spectra of MNP magnetization are characterized for temperature determination [11,12]. In addition, the phase lag of an MNP harmonic is measured to realize MNP thermometry in a high-frequency AC magnetic field [13,14]. However, all the approaches measure the integral, average temperature of an MNP sample, but not the temperature distribution.

In 2005, magnetic particle imaging (MPI) was first introduced to directly measure the spatial distribution of MNP concentration [15]. To realize MPI, a gradient DC magnetic field and AC excitation magnetic fields are applied for spatial encoding and the excitation of magnetic particles. Ideally, magnetic particles can only respond to the AC magnetic fields in a field free point (FFP) or field free line (FFL), where the DC magnetic field is zero. The magnetic particles that are far away from the FFP or FFL get saturated and do not respond to the AC magnetic fields. With this idea, several approaches reported the realization of MPI with magnetic scanning of a field free point or field free line [15,16,17,18,19,20,21]. Then, MPI was extended towards a functional imaging of MNP mobility or the viscosity of the surrounding matrix [22,23]. The approach of multicolor MPI was also used to measure the spatial distribution of MNP temperature, which has the potential of in-vivo temperature imaging [24]. Besides the electrical-scanning approaches, mechanical scanning and a multichannel system were presented to measure the spatial distribution of MNP concentration [25,26]. Recently, a scanning magnetic particle spectrometer (SMPS) was designed to simultaneously measure the spatial distributions of MNP concentration and temperature with a defined point spread function (PSF) and a proper deconvolution method. It has demonstrated the feasibility of the SMPS for noninvasive temperature imaging [27]. However, the spatial and temperature resolutions in MNP temperature imaging, as well as their dependence on the deconvolution or reconstruction, have not yet been quantitatively explored.

This paper quantitatively investigates the spatial and temperature resolutions of MNP temperature imaging. A multiline phantom, consisting of several parallel lines filled with MNPs, with different distances between two adjacent lines, is filled with an MNP sample for experiments. The first and third harmonics of the MNP sample are measured with a custom-built SMPS to simultaneously image MNP concentration and temperature. In this paper, the first harmonic amplitude is used to show the concentration image, while the harmonic ratio *R*_3rd/1st_, independent of MNP concentration, is used to determine the temperature image [28,29]. A deconvolution method based on a defined PSF is used to independently deconvolve the measured harmonics to improve the spatial resolution of MNP temperature imaging. A modulation transfer function (MTF) from the MNP concentration image is used to quantitatively characterize the spatial resolution, while the standard deviation of measured temperatures from the MNP temperature image is characterized to determine the temperature resolution. The spatial and temperature resolutions of the MNP temperature imaging, as well as their dependence on the deconvolution, are presented and discussed.

## 2. Experimental Description

### 2.1. Experimental Methods for MNP Temperature Imaging

In an AC magnetic field, MNP harmonics are dependent on MNP concentration and temperature. The models for the description of MNP magnetization, such as the static Langevin function and Fokker-Planck equation (FPE), indicate that MNP concentration has a linear relationship with MNP harmonic amplitudes, whereas MNP temperature has a nonlinear relationship with MNP harmonics in a small temperature range, for example, from 295 to 325 K [28,29]. Thus, the harmonic ratio, such as the ratio of the 3rd to the 1st harmonic, *R*_3rd/1st_, is independent of MNP concentration, but only dependent on MNP temperature. Therefore, each harmonic can be used to realize MNP concentration measurement whereas the harmonic ratio of two harmonics can be used to realize temperature determination independent of concentration.

In this paper, the spatial distributions of the 1st and 3rd harmonics are measured with a custom-built SMPS. The sensitivity profile of the pickup coil, *s*(*x*, *y*), defined as the PSF of the imaging system, is independent of MNP magnetic properties, but dependent on the geometry of the pickup coil, such as the diameter of the pickup coil, and the distance between the pickup coil and the MNP sample. Then, the measured 1st and 3rd harmonics *u*_1_(*x*, *y*) and *u*_3_(*x*, *y*) are, respectively, a convolution of the PSF *s*(*x*, *y*) and the 1st and 3rd harmonics *M*_1_(*x*, *y*, *c*, *T*) and *M*_3_(*x*, *y*, *c*, *T*) generated by local MNPs [27]:

*u*_1_(*x*, *y*) = *ω*·*s*(*x*, *y*) * *M*_1_(*x*, *y*, *c*, *T*) (1)

*u*_3_(*x*, *y*) = 3*ω* ·*s*(*x*, *y*) * *M*_3_(*x*, *y*, *c*, *T*) (2)

where * means convolution, *M*_1_(*x*, *y*, *c*, *T*) and *M*_3_(*x*, *y*, *c*, *T*) depend on MNP concentration, *c*, and temperature, *T*, in a spatial position (*x*, *y*), and *ω* is the angular frequency of the applied AC magnetic field. A deconvolution method based on the PSF enables the measurements of the spatial distributions of the 1st and 3rd harmonics generated by local MNPs. In this paper, an iterative reconstruction method called a simultaneous algebraic reconstruction technique (SART) is used to independently deconvolve the measured images of the 1st and 3rd harmonics, which independently minimizes ${\left\|GM_{1}-u_{1}/\omega \right\|}^{2}+\lambda {\left\|M_{1}\right\|}^{2}$ and ${\left\|GM_{3}-u_{3}/3\omega \right\|}^{2}+\lambda {\left\|M_{3}\right\|}^{2}$ with noise-suppressing features [30]. Herein, *G* is the system matrix, calculated from *s*(*x*, *y*), and λ is a regularization parameter. The estimated image of 1st and 3rd harmonics *M*_1_*^k^*(*x, y*) and *M*_3_*^k^*(*x, y*) (*k* = 1, 2, 3, …., *K*_max_) are, respectively, calculated from

(3) $$M_{1}^{k}(x,y)=M_{1}^{k-1}(x,y)+\lambda V^{-1}G\prime W^{-1}\left[u_{1}/\omega -GM_{1}^{k-1}(x,y)\right]\;$$

(4) $$M_{3}^{k}(x,y)=M_{3}^{k-1}(x,y)+\lambda V^{-1}G\prime W^{-1}\left[u_{3}/3\omega -GM_{3}^{k-1}(x,y)\right]\;$$

where *V* and *W* are diagonal matrices with row and column sums of *G* in the diagonal. The fixed regularization parameter *λ* = 1.9 is a constant in the iteration [30]. The initial guess *M_i_*^0^(*x, y*) is set to zero. In the implementation of the iterative deconvolution, the maximum iteration *K*_max_ is set to stop the iteration. MNP concentration imaging can be realized with either *M*_1_ or *M*_3_, whereas MNP temperature imaging can be achieved with the harmonic ratio *R*_3rd/1st_ = *M*_3_/*M*_1_.

### 2.2. Experimental Setup and Materials

**Figure 1:**
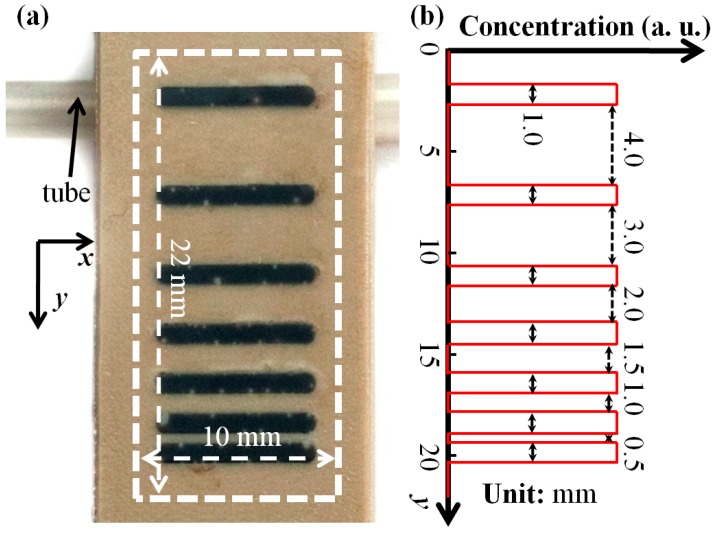
(**a**) Photo of the multiline phantom; (**b**) schematic of the concentration-versus-*y* curve.

A multiline phantom with different distances between two adjacent lines is filled with an MNP suspension for phantom experiments with a custom-built SMPS. Figure 1a shows the multiline phantom filled with MNPs. Each line in the phantom has a length of 8 mm, a width of 1 mm and a depth of 1.5 mm. Figure 1b shows a schematic of the concentration-versus-*y* curve. The distances between two adjacent lines are 0.5, 1.0, 1.5, 2.0, 3.0 and 4.0 mm, respectively. The experimental MNP sample is SHP-30, purchased from Ocean NanoTech. Ltd. Corp. (San Diego, CA, USA), consisting of Fe_3_O_4_ single-core nanoparticles with a nominal core diameter of 30 nm, a coating of monolayer oleic acid and monolayer amphiphilic polymer, and concentration of 5 mg/mL (Fe). The MNPs are characterized by a custom-built AC susceptibility system, showing a peak in the imaginary part of the complex AC susceptibility at about 3 kHz. It corresponds to a median hydrodynamic diameter of about 51 nm, which fits very well with optical approaches [31]. In addition, the fitting between the experiments and theoretical analysis without taking into account the dipolar interaction also means that the dipolar interaction is negligible. Thus, the dipolar interactions between experimental MNPs are not taken into account in this paper. The SMPS uses a Helmholtz coil for the generation of an AC magnetic field and a gradiometric pickup coil with a diameter of about 2.5 mm for the measurement of MNP magnetization. The details of the SMPS design are presented in Reference [27].

## 3. Results and Discussion

### 3.1. Calibration of Temperature-Dependent Harmonic Ratio

In our previous study, the static Langevin function was used to describe the temperature-dependent harmonic ratio of MNP magnetization while ignoring MNP dynamics. It showed that the harmonic ratio *R*_3rd/1st_ decreased with increasing temperature. However, the dynamics of SHP-30 in an AC magnetic field with amplitude of 10 mT and frequency of 2004 Hz cannot be ignored, causing the inapplicability of the static Langevin function. In this paper, the temperature-dependent harmonic ratio *R*_3rd/1st_ is calibrated in advance with the SMPS by changing the sample temperature. A sample of SHP-30 with a line with a width of 2 mm, a length of 8 mm and a depth of 1.5 mm is used to perform the calibration experiment. A water tube with temperature-controlled cycling water is placed below the MNP sample to change the sample’s temperature. An infrared thermometer VarioCAM, purchased from InfraTec GmbH (Dresden, Germany), is used to measure the sample’s temperature, whereas the SMPS is used to measure the harmonic ratio *R*_3rd/1st_.

**Figure 2:**
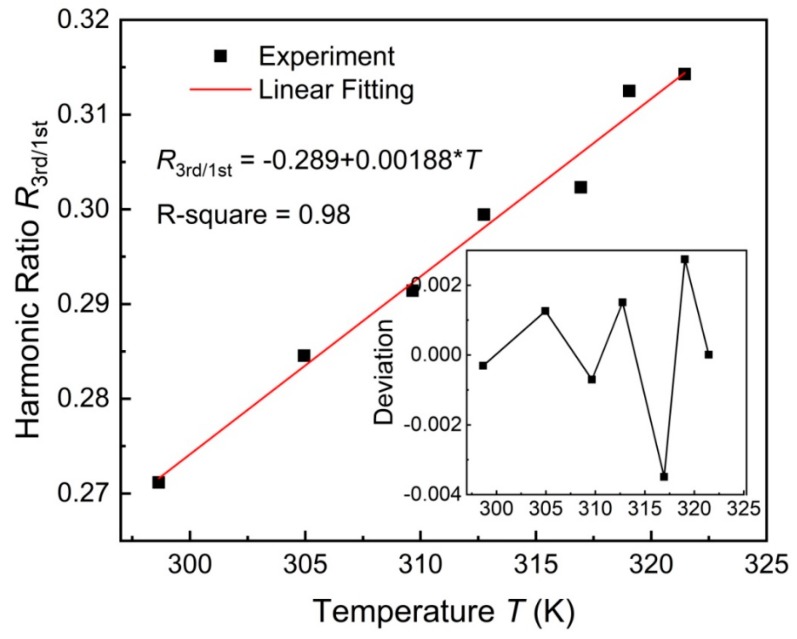
Harmonic ratio *R*_3rd/1st_ versus temperature *T*. The applied AC magnetic field has amplitude of 10 mT and frequency of 2004 Hz. The inset shows the deviation between the experimental and linear fitting harmonic ratios. Symbols represent experimental results whereas solid line is a linear regression curve.

Figure 2 shows the harmonic ratio *R*_3rd/1st_ versus temperature, which indicates that the harmonic ratio *R*_3rd/1st_ increases with increasing temperature. This phenomenon shows the opposite behaviour expected from the static Langevin function [12]. In a sufficiently high-frequency AC magnetic field, MNP dynamics play a significant role in the MNP harmonics. For SHP-30, dominated by Brownian relaxation *τ*_B_, the FPE indicates that the dynamic MNP magnetization is significantly affected by *ωτ*_B_(*T*), with the angular frequency ω of the applied magnetic field and the temperature-dependent Brownian relaxation time *τ*_B_(*T*). With increasing temperature, the Brownian relaxation time *τ*_B_(*T*) significantly decreases due to a higher thermal energy and a lower viscosity. It means that MNPs can rotate faster to follow the excited AC magnetic field, thus strengthening the MNP magnetization, especially higher harmonics [32,33]. Thus, a higher temperature leads to a higher harmonic ratio, as shown in Figure 2. In the small temperature range from 298 to 322 K, the temperature-dependent harmonic ratio *R*_3rd/1st_ is fitted with a linear equation (see solid line in Figure 2). The coefficient of determination *R*-square applying linear regression is 0.98, while the maximum deviation between the experimental and fitting harmonic ratio is below 0.004 (see the inset in Figure 2), meaning that the linear equation can describe the temperature-dependent harmonic ratio in the given temperature range. Figure 2 indicates that the temperature sensitivity d*R*_3rd/1st_/d*T* of the harmonic ratio is 0.00188 K^−1^.

### 3.2. MNP Temperature Imaging

**Figure 3:**
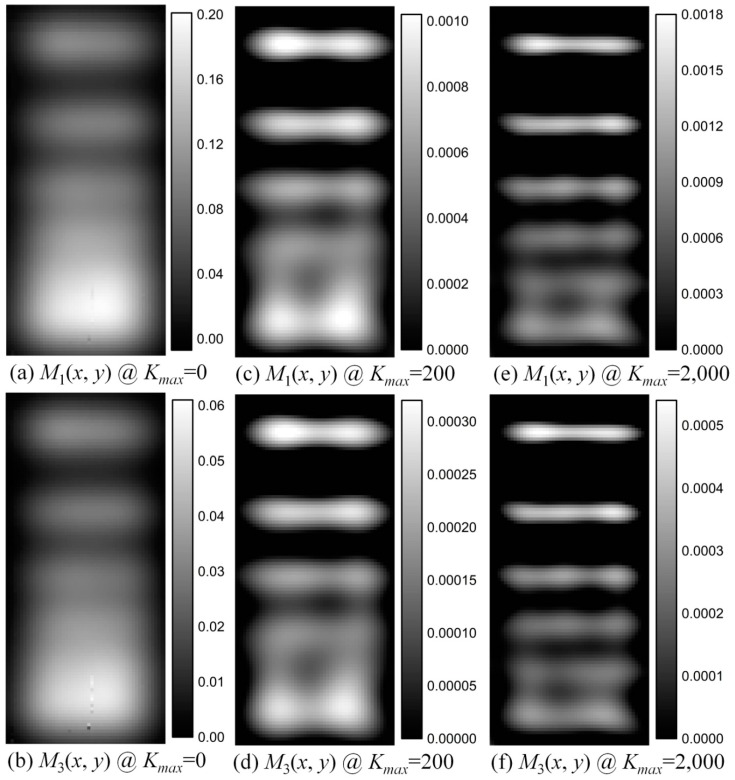
(**a**,**c**,**e**) show the measured and deconvolved spatial distributions of the 1st harmonics for K_max_ = 0, 200 and 2000, respectively. (**b**,**d**,**f**) show the measured and deconvolved spatial distributions of the 3rd harmonics for *K*_max_ = 0, 200 and 2000, respectively. The scanning FOV is 10 mm × 22 mm with a scanning step of 0.2 mm. The images of the 1st and 3rd harmonics for *K*_max_ = 0 represent measured (undeconvolved) ones.

The 1st and 3rd harmonics are measured with the SMPS to realize MNP concentration and temperature imaging. The scanning field of view (FOV) is 10 mm × 22 mm in *x*- and *y*-directions with a scanning step of 0.2 mm. The scanning time amounts to about 15 min. A water tube with water cycled by a pump was placed below the first line to change the temperature profile of the phantom, as shown in Figure 1a. The water temperature was controlled in a water bath at about 346 K (about 73 °C). An AC magnetic field with amplitude of 10 mT and frequency of 2004 Hz is applied to excite the MNPs. Figure 3 show the measured and deconvolved spatial distributions of the 1st (the first row) and 3rd (the second) harmonics for different *K_max_* values. Note that *K_max_* = 0 means that the harmonics are the measured ones without deconvolution. Thus, the images in the first column in Figure 3 are measured data without deconvolution. Figure 3 indicates that the measured image can only resolve two lines with a distance of 3 mm, whereas the deconvolved images for *K_max_* = 200 (2000) can resolve two lines with a distance of 2 mm (1.5 mm) with deconvolution. Thus, the deconvolution improves the spatial resolution.

**Figure 4:**
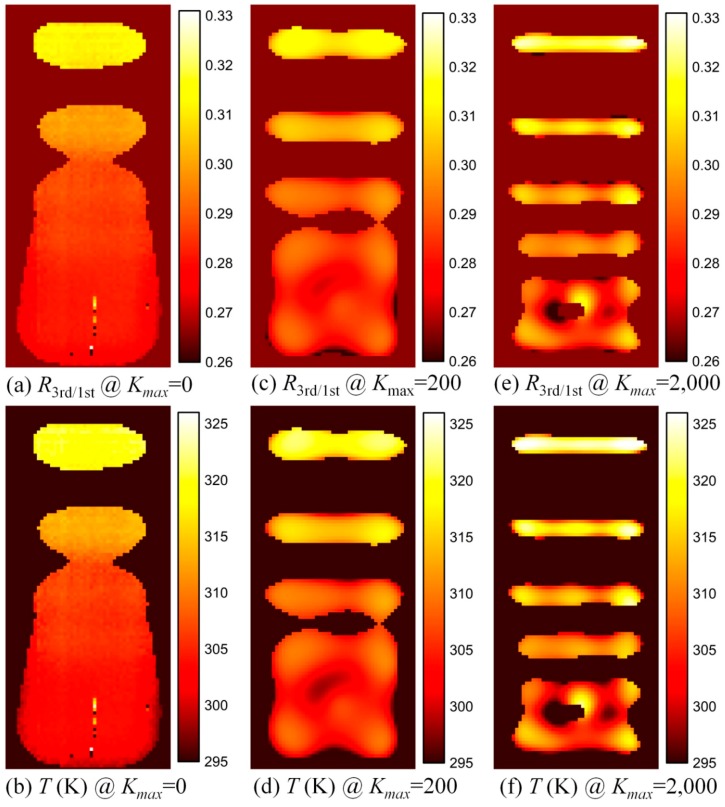
Deconvolved spatial distributions of harmonic ratio (the first row) and temperature (the second row) for different K_max_ values. (**a**,**c**,**e**) show the deconvolved spatial distributions of harmonic ratio, whereas (**b**,**d**,**f**) show the deconvolved spatial distributions of temperature.

With the deconvolved spatial distributions of the 1st and 3rd harmonics, the spatial distributions of harmonic ratio *R*_3rd/1st_ for different *K_max_* values are depicted in Figure 4. Note that MNP temperature imaging is not able to provide temperature information at a position where there are no MNPs. Thus, a threshold *γ* = 0.3 is applied in the calculation of the harmonic ratio *R*_3rd/1st_ in this paper. The harmonic ratio *R*_3rd/1st_ in a position where *M*_1_(*x*, *y*) <*γ·M*_max_, meaning there are no MNPs in the position, was set to 0.266 at a room temperature (about 295 K). Herein, *M*_max_ is the maximum value of the measured or deconvolved 1st harmonic. The first row in Figure 4 shows the deconvolved images of the harmonic ratio *R*_3rd/1st_ for different *K_max_* values. From the spatial distribution of the harmonic ratio, MNP temperature imaging is realized with the calibration curve of harmonic ratio *R*_3rd/1st_ versus temperature (see Figure 2). The second row in Figure 4 shows the corresponding deconvolved temperature images, which clearly show that the first line of the phantom has a higher temperature than the others. Some gross errors, caused by the instability of the SMPS, can be found in the measured temperature image without deconvolution. Furthermore, the temperature, as expected, decreases with increasing distance between the phantom line and the hot-water tube.

### 3.3. Spatial Resolution

**Figure 5:**
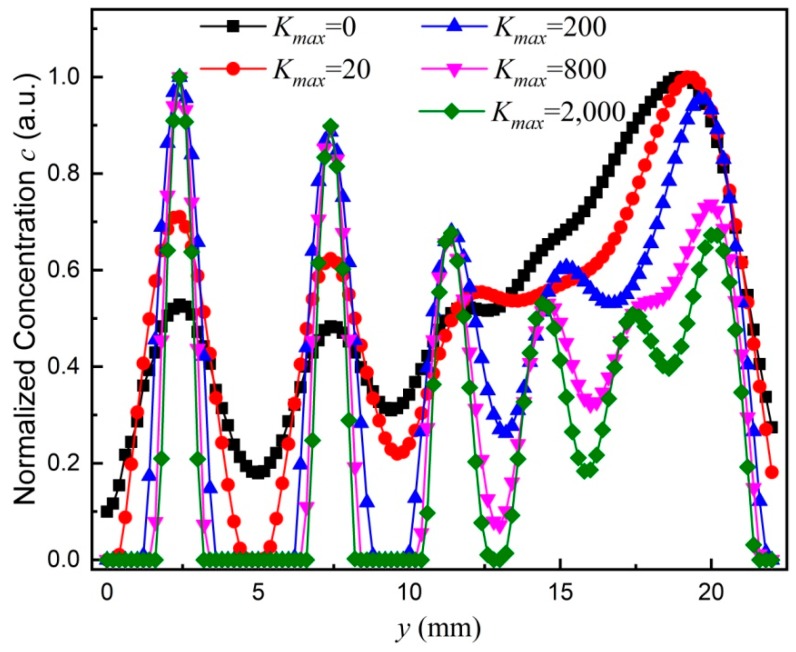
Normalized concentration versus *y* for *K_max_* values. Symbols represent experimental results whereas solid lines are guides to the eye.

Different *K_max_* values are applied in the deconvolution to investigate the effect on the spatial resolution. The 1st harmonics are used to characterize the MNP concentration image. Figure 5 displays the concentration *c* versus *y* position (*c*-*y* curve) for different *K_max_* values. Herein, the *c*-*y* curve is averaged over different *x* positions. With *K_max_* = 0 and 20, the two lines with a distance of 3 mm can be resolved. Only four peaks are discernible from the *c*-*y* curve. With *K_max_* = 200 and 800, five peaks are discernible, whereas the two lines with a distance of 2.0 mm can be resolved. With *K_max_* = 2000, six peaks are discernible, whereas the two lines with a distance of 1.0 mm can be resolved. However, the deconvolved image with an even higher *K_max_* cannot resolve the two lines with a distance of 0.5 mm.

**Figure 6:**
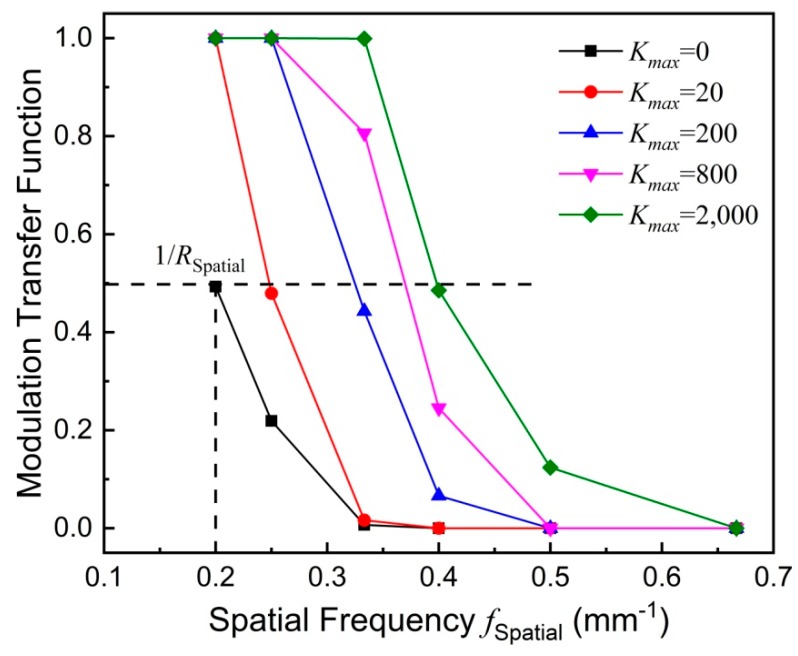
Modulation transfer function versus spatial frequency for different *K_max_* values. Symbols represent experimental results whereas solid lines are guides to the eye.

**Figure 7:**
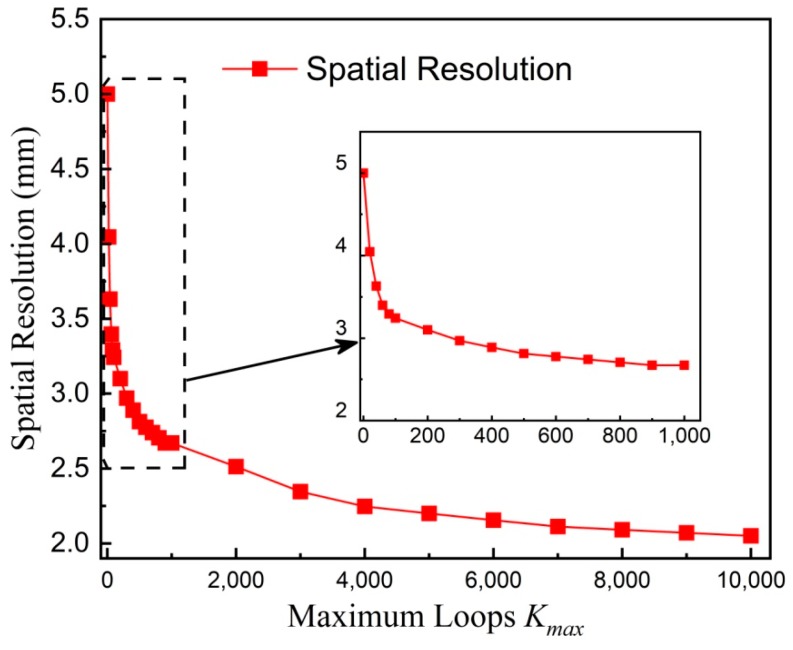
Spatial resolution versus *K_max_*. The inset is locally zoomed-in data. Symbols represent experimental results whereas the solid line is a guide to the eye.

A modulation transfer function *MTF*(*f*_spatial_) is calculated from the *c*-*y* curve for a quantitative estimation of the spatial resolution [34,35]. Herein, *f*_Spatial_ represents the spatial frequency. The MTF is defined as *MTF*(*f*_Spatial_) = (*c*_max_ − *c*_min_)/(*c*_max_ + *c*_min_), where *c*_max_ (*c*_min_) is the maximum (minimum) value of the *c*–*y* curve at a certain spatial frequency *f*_Spatial_. For instance, a spatial frequency *f*_Spatial_ of 0.2 mm^−1^ is calculated by 1/(*d* + *w*) for a distance *d* = 4 mm between two lines and a line width *w* = 1 mm. Figure 6 shows *MTF*(*f*_Spatial_) versus *f*_Spatial_, calculated from the *c*-*y* curve shown in Figure 5. The spatial resolution *R*_Spatial_ is defined as the value at which *MTF*(1/*R*_Spatial_) equals 0.5. It indicates that 1/*R*_Spatial_ increases with increasing *K_max_*, meaning that the spatial resolution *R*_spatial_ is improved. A cubic interpolation is used to calculate 1/*R*_spatial_. With this definition of spatial resolution, Figure 7 presents the spatial resolution versus *K_max_*. With *K_max_* = 0, the spatial resolution *R*_Spatial_ amounts to about 5 mm. With increasing *K_max_* to 1000, the spatial resolution *R*_Spatial_ is significantly improved to about 2.6 mm. With *K_max_* greater than 4000, the spatial resolution *R*_Spatial_ is getting saturated. For *K_max_* of 10,000 or greater, the spatial resolution *R*_Spatial_ is about 2 mm. Note that the distance between the pickup coil and the phantom is larger than 1.75 mm. Therefore, the highest achievable spatial resolution is about 2 mm.

### 3.4. Temperature Resolution

**Figure 8:**
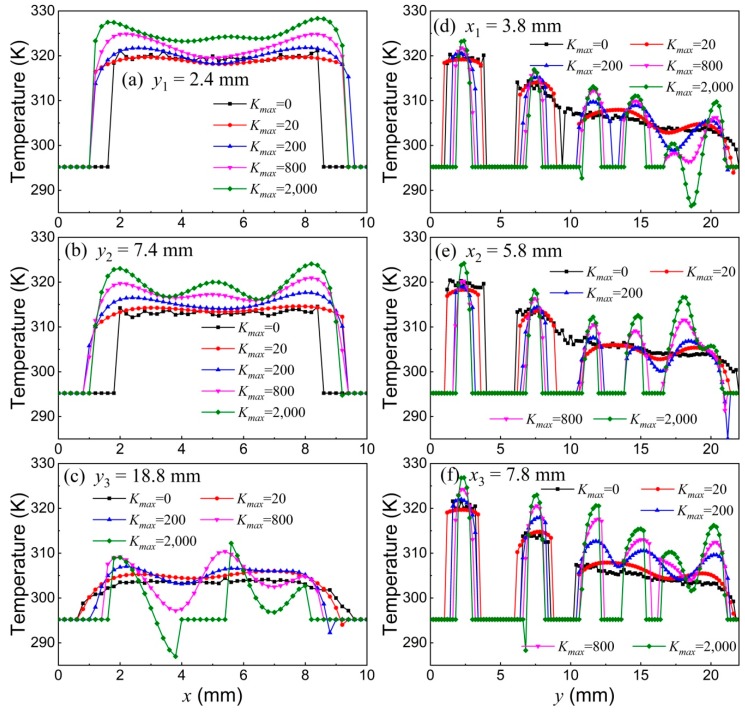
(**a**–**c**) Measured temperatures versus *x* for different *K_max_* values at different *y* positions. (**d**–**f**) Measured temperatures versus *y* for different *K_max_* values at different *x* positions. Symbols represent experimental results whereas solid lines are guides to the eye.

Figure 8a–c shows the measured temperature *T* versus *x* position (*T*-*x* curve) at three different *y* positions, whereas Figure 8d–f shows the measured temperature *T* versus *y* position (*T*-*y* curve) at three different *x* positions for different *K_max_*. The comparison of Figure 8a–c indicates that the measured temperature *T*, as expected, decreases with increasing *y* (increasing the distance between the MNP sample and the hot-water tube), which can also be seen from the *T*-*y* curves in Figure 8d–f. Figure 8a–c indicates that the measured temperatures at different *x* positions but at the same *y* position are, as expected, the same. For *K_max_* = 0, the fluctuation in the measured temperatures mainly comes from the measurement noise, as shown by the black solid line in Figure 8. With increasing *K_max_*, both the *T*-*x* and *T*-*y* curves show pronounced oscillations. Therefore, a greater *K_max_* value qualitatively results in a worse temperature resolution.

**Figure 9:**
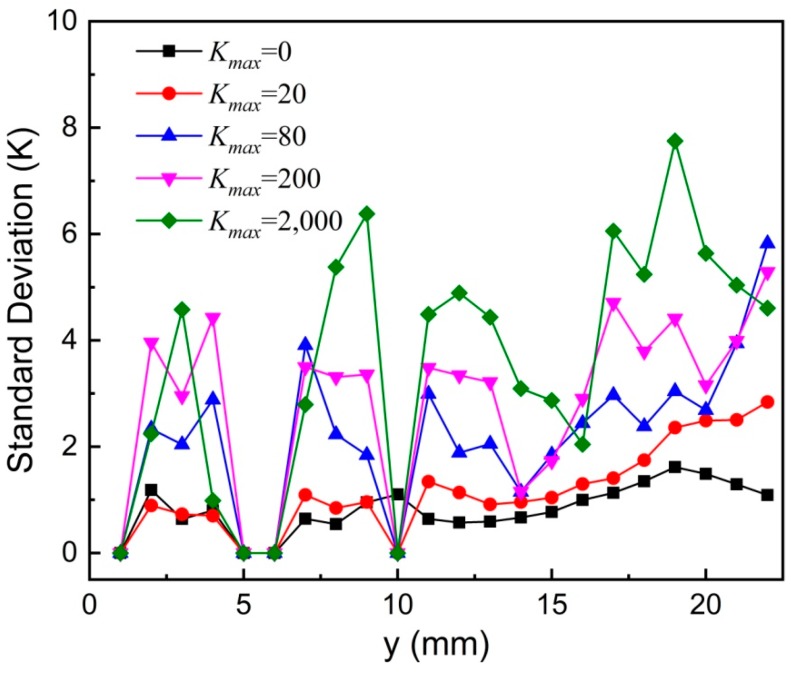
Standard deviation of measured temperature versus *y* position for different *K_max_* values. Symbols represent experimental results whereas solid lines are guides to the eye.

In principle, the MNP temperatures at different *x* positions but at the same *y* position are expected to be the same, which can be seen from the *T*-*x* curves for *K_max_* = 0. In addition, the temperatures at five adjacent *y* positions (in a 1-mm area in the *y* direction) are almost the same. Thus, the standard deviation *δ*(*y*) is calculated from the measured temperatures at different *x* positions and at five adjacent *y* positions excluding the temperatures set to room temperature. The standard deviation *δ*(*y*) versus *y* position is depicted in Figure 9 for different *K_max_* values. It indicates that the standard deviation *δ*(*y*) gradually increases with increasing *K_max_*, representing a worse temperature resolution at a greater *K_max_*.

**Figure 10:**
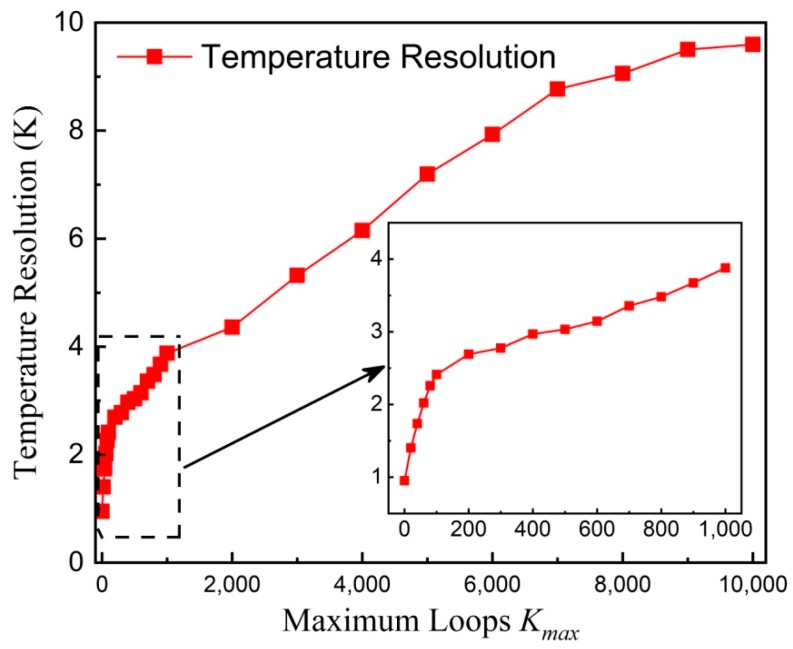
Temperature resolution versus *K_max_*. The inset is locally zoomed-in data. Symbols represent experimental results whereas the solid line is a guide to the eye.

To quantitatively investigate the temperature resolution, it is approximated by the standard deviation *δ*(*y*) averaged over different *y* positions for different *K_max_*. Herein, the standard deviation *δ*(*y*) equaling 0 is excluded in the calculation of the average value of *δ*(*y*). Figure 10 shows the temperature resolution versus *K_max_*. As can be seen, the temperature resolution gets worse with increasing *K_max_*. For *K_max_* = 0, the temperature resolution is about 1.0 K, which mainly comes from the measurement noise and the instability of the controlled water temperature. Note that the temperature resolution without deconvolution is worse than that of about 0.2 K reported in [27], which is caused by the different measurement times. In this paper, the whole measurement for an image is about 15 min whereas it is about 3 h, meaning that there are more average times, in [27]. For *K_max_* = 100, the temperature resolution is about 2.4 K. Further increasing *K_max_*, the temperature resolution gets worse and worse. With *K_max_* = 10,000, the temperature resolution, originating from the deconvolution-based oscillation, is about 9.6 K.

## 4. Conclusions

The spatial and temperature resolutions are key parameters in MNP temperature imaging. This paper quantitatively investigates the temperature and spatial resolutions of MNP temperature imaging. A multiline phantom with different distances between two adjacent lines is used to perform experiments. The 1st and 3rd harmonics of the MNPs are measured with a custom-built scanning magnetic particle spectrometer. The 1st and 3rd harmonics are used to determine the concentration image by the reconstruction method with the measured point spread function, while the harmonic ratio is applied to determine the temperature image independent of concentration. An iterative deconvolution method is performed to improve the spatial resolution. A modulation transfer function is calculated from the concentration image for the spatial resolution, whereas the average standard deviation of measured temperatures is calculated for the temperature resolution. The influence of the deconvolution on the spatial and temperature resolutions is quantitatively investigated by changing the iterative loops in the deconvolution. Experimental results indicate that the deconvolution increases the spatial resolution but worsens the temperature resolution due to deconvolution-based oscillation. It demonstrates that there is a trade-off between the spatial and temperature resolutions.

## References

[1] Vreugdenburg T.D., Willis C.D., Mundy L., Hiller J.E. (2013). A systematic review of elastography, electrical impedance scanning, and digital infrared thermography for breast cancer screening and diagnosis. Breast Cancer Res. Treat..

[2] Acharya U.R., Ng E.Y.-K., Tan J.-H., Sree S.V. (2012). Thermography based breast cancer detection using texture features and support vector machine. J. Med. Syst..

[3] Thiesen B., Jordan A. (2008). Clinical applications of magnetic nanoparticles for hyperthermia. Int. J. Hyperth..

[4] Johannsen M., Gneveckow U., Thiesen B., Taymoorian K., Cho C.H., Waldöfner N., Scholz R., Jordan A., Loening S.A., Wust P. (2007). Thermotherapy of prostate cancer using magnetic nanoparticles: Feasibility, imaging, and three-dimensional temperature distribution. Eur. Urol..

[5] Pradhan P., Giri J., Rieken F., Koch C., Mykhaylyk O., Döblinger M., Banerjee R., Bahadur D., Plank C. (2010). Targeted temperature sensitive magnetic liposomes for thermo-chemotherapy. J. Control. Release.

[6] Kneidl B., Peller M., Winter G., Lindner L.H., Hossann M. (2014). Thermosensitive liposomal drug delivery systems: State of the art review. Int. J. Nanomed..

[7] Li L., Hagen T.L.T., Schipper D., Wijnberg T.M., van Rhoon G.C., Eggermont A.M., Lindner L.H., Koning G.A. (2010). Triggered content release from optimized stealth thermosensitive liposomes using mild hyperthermia. J. Control. Release.

[8] Zhong J., Liu W., Du Z., de Morais P.C., Xiang Q., Xie Q. (2012). A noninvasive, remote and precise method for temperature and concentration estimation using magnetic nanoparticles. Nanotechnology.

[9] Zhong J., Liu W., Jiang L., Yang M., Morais P.C. (2014). Real-time magnetic nanothermometry: The use of magnetization of magnetic nanoparticles assessed under low frequency triangle-wave magnetic fields. Rev. Sci. Instrum..

[10] Zhong J., Liu W., Kong L., Morais P.C. (2014). A new approach for highly accurate, remote temperature probing using magnetic nanoparticles. Sci. Rep..

[11] Weaver J.B., Rauwerdink A.M., Hansen E.W. (2009). Magnetic nanoparticle temperature estimation. Med. Phys..

[12] Zhou M., Zhong J., Liu W., Du Z., Huang Z., Yang M., Morais P.C. (2015). Study of magnetic nanoparticle spectrum for magnetic nanothermometry. IEEE Trans. Magn..

[13] He L., Liu W., Xie Q., Pi S., Morais P. (2015). A fast and remote magnetonanothermometry for a liquid environment. Meas. Sci. Technol..

[14] Garaio E., Collantes J.-M., Garcia J.A., Plazaola F., Sandre O. (2015). Harmonic phases of the nanoparticle magnetization: An intrinsic temperature probe. Appl. Phys. Lett..

[15] Gleich B., Weizenecke J. (2005). Tomographic imaging using the nonlinear response of magnetic particles. Nature.

[16] Sattel T.F., Knopp T., Biederer S., Gleich B., Weizenecker J., Borgert J., Buzug T.M. (2008). Single-sided device for magnetic particle imaging. J. Phys. D Appl. Phys..

[17] Vogel P., Ruckert M.A., Klauer P., Kullmann W.H., Jakob P.M., Behr V.C. (2014). Traveling wave magnetic particle imaging. IEEE Trans. Med. Imaging.

[18] Goodwill P.W., Conolly S.M. (2010). The X-space formulation of the magnetic particle imaging process: 1-D signal, resolution, bandwidth, SNR, SAR, and magnetostimulation. IEEE Trans. Med. Imaging.

[19] Weizenecker J., Gleich B., Borgert J. (2008). Magnetic particle imaging using a field free line. J. Phys. D Appl. Phys..

[20] Goodwill P.W., Konkle J.J., Zheng B., Saritas E.U., Conolly S.M. (2012). Projection x-space magnetic particle imaging. IEEE Trans. Med. Imaging.

[21] Pi S., Liu W., Jiang T. (2018). Real-time and quantitative isotropic spatial resolution susceptibility imaging for magnetic nanoparticles. Meas. Sci. Technol..

[22] Rahmer J., Halkola A., Gleich B., Schmale I., Borgert J. (2015). First experimental evidence of the feasibility of multi-color magnetic particle imaging. Phys. Med. Biol..

[23] Viereck T., Kuhlmann C., Draack S., Schilling M., Ludwig F. (2017). Dual-frequency magnetic particle imaging of the Brownian particle contribution. J. Magn. Magn. Mater..

[24] Stehning C., Gleich B., Rahmer J. (2016). Simultaneous magnetic particle imaging (MPI) and temperature mapping using multi-color MPI. Int. J. Magn. Part. Imaging.

[25] Richter H., Kettering M., Wiekhorst F., Steinhoff U., Hilger I., Trahms L. (2010). Magnetorelaxometry for localization and quantification of magnetic nanoparticles for thermal ablation studies. Phys. Med. Biol..

[26] Fodil K., Denoual M., Dolabdjian C. (2016). Experimental and analytical investigation of a 2-D magnetic imaging method using magnetic nanoparticles. IEEE Trans. Magn..

[27] Zhong J., Schilling M., Ludwig F. (2018). Magnetic nanoparticle temperature imaging with a scanning magnetic particle spectrometer. Meas. Sci. Technol..

[28] Zhong J., Dieckhoff J., Schilling M., Ludwig F. (2016). Influence of static magnetic field strength on the temperature resolution of a magnetic nanoparticle thermometer. J. Appl. Phys..

[29] Zhong J., Schilling M., Ludwig F. (2017). Magnetic nanoparticle thermometry independent of Brownian relaxation. J. Phys. D Appl. Phys..

[30] Andersen H., Kak A.C. (1984). Simultaneous algebraic reconstruction technique (SART): A superior implementation of the ART algorithm. Ultrason. Imaging.

[31] Dieckhoff J., Eberbeck D., Schilling M., Ludwig F. (2016). Magnetic-field dependence of Brownian and Neel relaxation times. J. Appl. Phys..

[32] Draack S., Viereck T., Kuhlmann C., Schilling M., Ludwig F. (2017). Temperature-dependent MPS measurements. Int. J. Magn. Part. Imaging.

[33] Wells J., Paysen H., Kosch O., Trahms L., Wiekhorst F. (2018). Temperature dependence in magnetic particle imaging. AIP Adv..

[34] Judy P. (1976). The line spread function and modulation transfer function of a computed tomographic scanner. Med. Phys..

[35] Knopp T., Biederer S., Sattel T.F., Erbe M., Buzug T.M. (2011). Prediction of the spatial resolution of magnetic particle imaging using the modulation transfer function of the imaging process. IEEE Trans. Med. Imaging.

